# Rates and risk factors of intrapedicular accuracy and cranial facet joint violation among robot-assisted, fluoroscopy-guided percutaneous, and freehand techniques in pedicle screw fixation of thoracolumbar fractures: a comparative cohort study

**DOI:** 10.1186/s12893-022-01502-5

**Published:** 2022-02-11

**Authors:** Ren-Jie Zhang, Lu-Ping Zhou, Hua-Qing Zhang, Peng Ge, Chong-Yu Jia, Cai-Liang Shen

**Affiliations:** Department of Orthopedics and Spine Surgery, The First Affiliated Hospital of Anhui Medical University, 210 Jixi Road, Hefei, 230022 Anhui China

**Keywords:** Robot-assisted, Fluoroscopy-guided percutaneous, Freehand, Pedicle screw placement accuracy, Proximal facet joint, Thoracolumbar fractures

## Abstract

**Background:**

Robot-assisted (RA) technique has been increasingly applied in clinical practice, providing promising outcomes of inserting accuracy and cranial facet joint protection. However, studies comparing this novel method with other assisted methods are rare, and the controversy of the superiority between the insertion techniques remains. Thus, we compare the rates and risk factors of intrapedicular accuracy and cranial facet joint violation (FJV) of RA, fluoroscopy-guided percutaneous (FP), and freehand (FH) techniques in the treatment of thoracolumbar fractures.

**Methods:**

A total of 74 patients with thoracolumbar fractures requiring pedicle screw instruments were retrospectively included and divided into RA, FP, and FH groups from June 2016 to May 2020. The primary outcomes were the intrapedicular accuracy and cranial FJV. The factors that affected the intrapedicular accuracy and cranial FJV were assessed using multivariate analyses.

**Results:**

The optimal intrapedicular accuracy of pedicle screw placement (Grade A) in the RA, FP, and FH groups was 94.3%, 78.2%, and 88.7%, respectively. This finding indicates no significant differences of RA over FH technique (P = 0.062) and FP technique (P = 0.025), but significantly higher accuracies of RA over FP (P < 0.001). In addition, the rates of proximal FJV in RA, FP, and FH groups were 13.9%, 30.8%, and 22.7%, respectively. RA had a significantly greater proportion of intact facet joints than the FP (P = 0.002). However, FP and FH (P = 0.157), as well as RA and FH (P = 0.035) showed significantly similar outcomes with respect to the proximal FJV. The logistic regression analysis showed that FP technique (OR = 3.056) was independently associated with insertion accuracy. Meanwhile, the age (OR = 0.974), pedicle angle (OR = 0.921), moderate facet joint osteoarthritis (OR = 5.584), and severe facet joint osteoarthritis (OR = 11.956) were independently associated with cranial FJV.

**Conclusion:**

RA technique showed a higher rate of intrapedicular accuracy and a lower rate of cranial FJV than FP technique, and similar outcomes to FH technique in terms of intrapedicular accuracy and cranial FJV. RA technique might be a safe method for pedicle screw placement in thoracolumbar surgery.

**Level of evidence:**

3

## Background

Thoracolumbar fractures account for most of the spine fractures, which can cause spinal instability and kyphosis, and often require surgical treatment. Pedicle screw reduction and internal fixation are the commonly used techniques. Since the launch of screw placement with freehand (FH) technique, numerous insertion techniques, such as fluoroscopy-guided percutaneous (FP), computer-assisted guidance, and robotic-assisted (RA) approaches have been introduced to achieve better surgical outcomes with increased inserting accuracy, reduced intraoperative blood loss, smaller incision, shorter surgical time, and better pain relief [1, 2]. However, the misplacement of pedicle screw might contribute to severe complications including persistent pain, neurological damage, vascular and muscular injuries, punctuation of trachea and pleura, and even spinal instability [3–5]. Besides, among the complications caused by malposition, the cranial facet joint violation (FJV) has been regarded as a crucial risk factor for adjacent segment degeneration (ASD) after pedicular fixation [3, 4, 6–8].

Recently, a novel insertion technique of RA method has been increasingly applied in clinical practice; it provides promising outcomes of inserting accuracy, ranging from 93.2 to 98.2% [9–15]. Many studies have reported that the RA technique remarkably improved the intrapedicular accuracy compared with the FP and FH techniques with advantages of automated planation, precise identification, and reduced manual errors in instrumental procedures [2, 6, 11, 12, 16, 17]. However, the clinical outcomes about the pedicle screw placement accuracy via different types of robots vary widely, and two randomized controlled trials (RCTs) with high quality have demonstrated that RA in intrapedicular accuracy showed no advantage over FH [13, 14]. Importantly, the RCT conducted by Ringel et al. [15] revealed that the RA method was associated with substantially reduced intrapedicular accuracy rate of 85% compared with the FH method with the rate of 93%.

Besides, many studies have found that the RA techniques resulted in lower cranial FJV rates (0–2.84%) than the FH methods (12.76–34.1%) [10, 13, 16, 18, 19]. Yang et al. performed a retrospective analysis and concluded that FP methods caused a significantly higher cranial FJV rate of 15.6% than RA methods of 5.1%. However, Hyun et al. [14] indicated that RA and FH techniques had similar cranial FJV occurrences of 100% and 99.29%, respectively, indicating that RA showed no superiority in facet joint protection over FH.

The thoracolumbar segments are in the transitional area of the facet joint anatomy, where the surfaces of facet joint change from the coronal to the sagittal planes. Thus, the thoracolumbar fractures might contribute to the dislocations in part of the facet joints and the shifts of normal anatomical landmarks for accurate placement of pedicle screw. Thus, the intrapedicular accuracy is reduced, and the rate of cranial FJV is increased. However, most literature regarding thoracolumbar fractures focused on the clinical efficiencies in different insertion techniques, but studies about the radiographic outcomes were rare [20–22]. Meanwhile, the superiority of different insertion techniques in intrapedicular accuracy and cranial FJV remains controversial. Therefore, the purpose of the study is to evaluate the rate and risk factors of the pedicle screw placement accuracy and cranial FJV among RA, FP, and FH techniques.

## Materials and methods

### Study design and patients

The retrospective study was approved by the hospital institutional review board. A total of 74 patients diagnosed of T11-L2 fractures with screw placement from T10 to L4 levels were included in the comparative study treated with robot-assisted (30 patients), fluoroscopy-guided percutaneous (14 patients), and freehand (30 patients) insertions during the period of June 2016 to May 2020. The diagnosis of T11 fracture accounted for 6.8%, and the T12, L1, and L2 fractures accounted for 27.0%, 45.9%, and 20.3%, respectively.

Patients that satisfy the following criteria were eligible: (1) patients suffering from T11-L2 fractures in the treatment of pedicle screw insertion with or without interbody fusion; (2) patients receiving postoperative 3D CT examination. The exclusion criteria were as follows: (1) prior surgery at the instrumental levels; (2) pathological fracture caused by tuberculosis or tumor; (3) incomplete data in the review case.

### Surgical techniques

#### Robot-assisted pedicle screw placement procedure

**Fig. 1:**
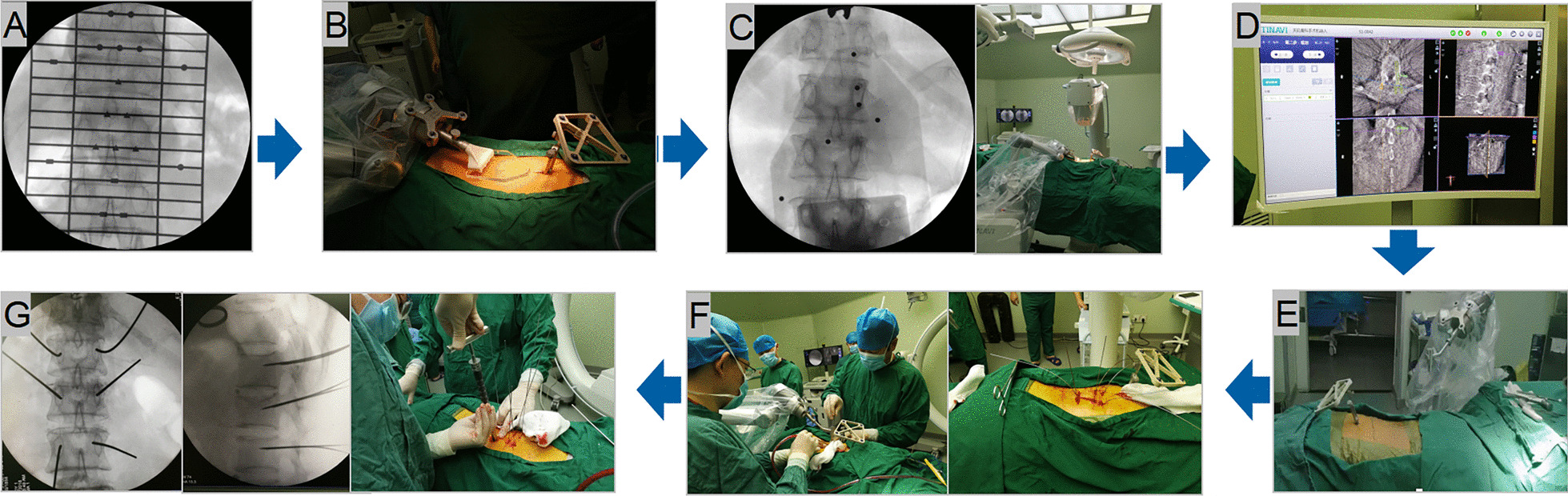
Robot-assisted pedicle screw placement procedure. **A** Confirm the position of the target vertebral body; **B** install the tracer and positioning ruler; **C** scan and collect the 3D image of the patient and transmit it to the robot workstation, and then the image is automatically registered; **D** preplan the entry point and insertion trajectory; **E** move the Robotic arm: simulation operation, execution operation; **F** insert the working sleeve, expand the soft tissue, and implant the guide wire; **G** confirm the position of the guide wire, and insert the screw along the guide wire

In the RA group, the TINAVI robot workstation was connected to the C-arm scanner. After scanning, the 3D image was transmitted to the TINAVI robot workstation for automated registration. According to the planned path, the final position of the robot arm was adjusted. Then, the working sleeve was inserted along the robot arm guide to touch the bony part of the vertebral body, and the guide wire was inserted with the electric drill along the direction of the working sleeve. The rod was placed after the insertions of the pedicle screw along the guide wire [12] (Fig. 1).

#### Fluoroscopy-guided pedicle screw placement procedure

In the FP group, under the frontal and lateral fluoroscopy with C-arm X-ray, the puncture needle was inserted through bilateral pedicle (3 and 9 o’clock positions in the right and left pedicle projections, respectively, and approximately 10°–15° inclination). The core of the puncture needle was removed after confirming that the position was satisfactory with fluoroscopies. Then, the guide wire was inserted, and the outer sheath of the puncture needle was withdrawn [23]. The methods of screw implantation were the same as in the RA group.

#### Freehand pedicle screw placement procedure

In the FH group, the paraspinal muscles were striped from the midline approach to both sides, and then the lateral edge of the articular process was exposed. The transverse process, facet joint, and isthmus were used as the landmarks for entry points in the pedicle screw placement. Then, the appropriately selected screws were inserted and secured with rods [24, 25].

### Outcome measures

**Fig. 2:**
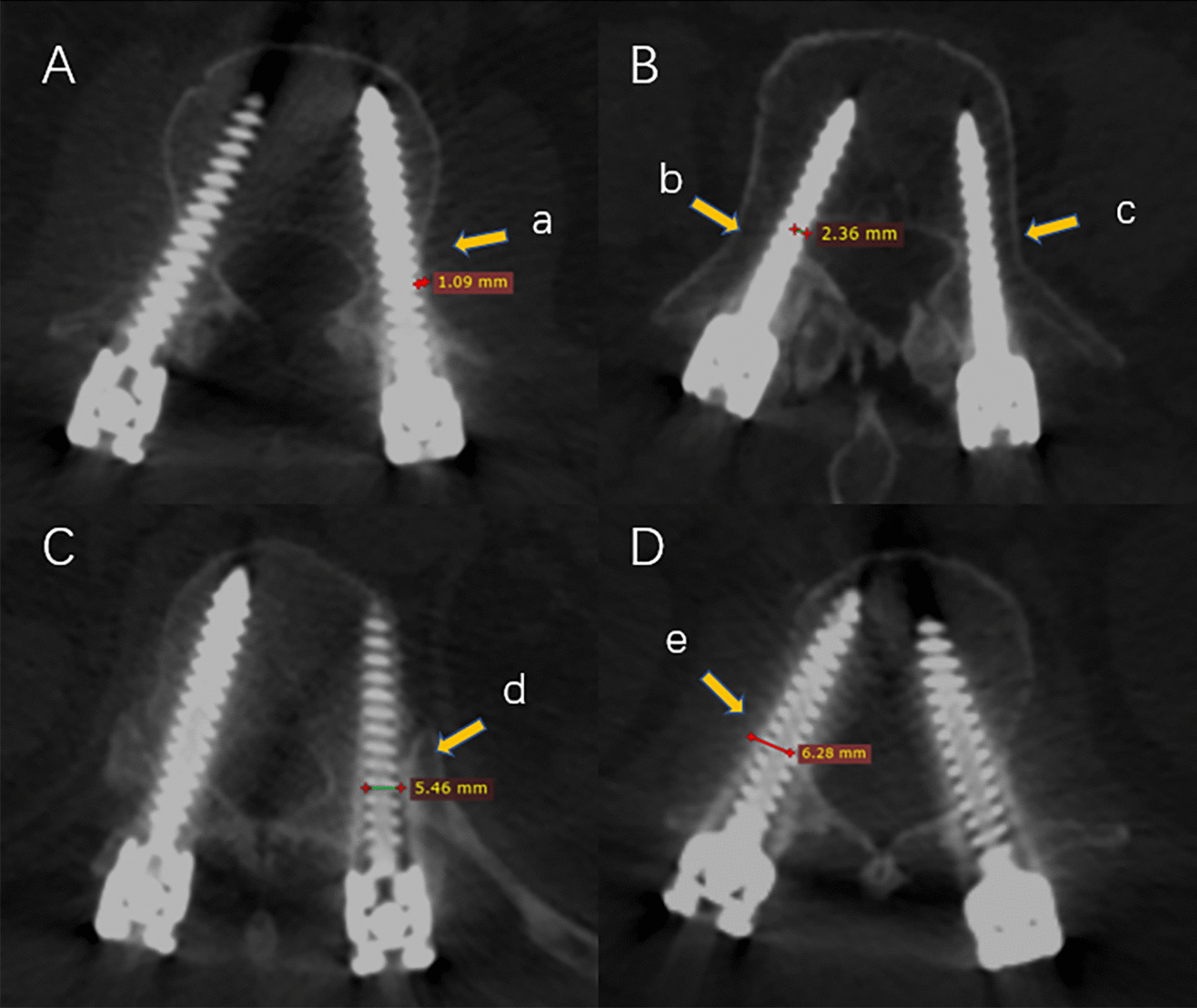
Intra-pedicular accuracy according to the Gertzbin–Robbins scale. **A** Grade B, cortical breach of 1.09 mm (a); **B** Grade C, cortical breach of 2.36 mm (b); and Grade A, screw position completely within the pedicle (c); **C** grade D, cortical breach of 5.46 mm (d); **D** Grade E, cortical breach of 6.28 mm (e)

**Fig. 3:**
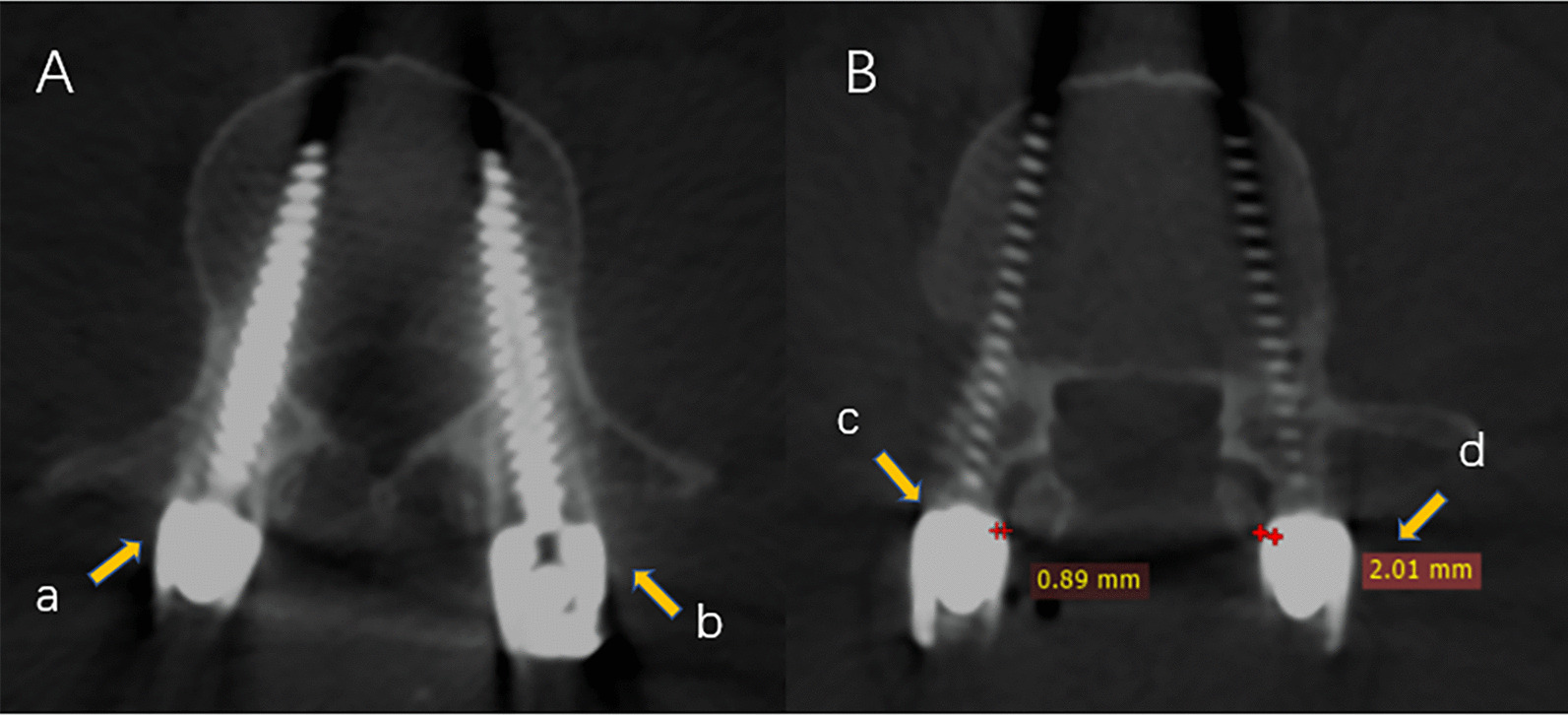
Proximal facet joint violation in accordance with the Babu scale. **A** Grade 0, screw not in facet, transverse position (a); and Grade 1, screw in lateral facet but not in facet articulation, transverse position (b); **B** Grade 2, penetrating facet articulation less than 1 mm, transverse position (c); and Grade 3, traveling within facet articulation, transverse position (d)

The primary outcomes were intrapedicular accuracy and facet joint violation, which were assessed in accordance with the Gertzbein and Robbins scale [26] (Fig. 2) and the Babu scale [19] (Fig. 3), respectively. Both grading systems were evaluated on the basis of postoperative 3D CT scans of the axial, coronal, and sagittal images. The Gertzbein and Robbins scale consisted of Grade A–E. Meanwhile, the graded A screws were considered perfect positions, graded A + B screws were clinically acceptable positions, and the graded C + D + E screws were regarded as malposition.

The secondary parameters were statistics of cases with the screw deviations of superior, inferior, medial, or lateral direction in the pedicle malposition, statistics on the screw parts of shaft, head, or rod causing cranial FJV, and complications including intraoperative revision due to screw malposition, postoperative revision due to screw malposition, wound infections, operation time, and estimated blood loss.

The following variables were also collected for the analysis of possible risk factors associated with intrapedicular accuracy and cranial FJV: age, gender, body mass index (BMI), pedicle screw placement technologies, pedicle angle, facet joint osteoarthritis, and distance from skin to insertion point. The facet joint osteoarthritis was evaluated with Pathria classification [27]. The radiographic data were independently assessed by two surgeons (R-J. Z. and H-Q. Z.) on the postoperative 3D CT images. If divergences existed between the two evaluators, then the third evaluator (C-L. S.) made the final decision.

### Statistical analysis

All statistical analyses were conducted using SPSS 23.0 software (IBM Corp., Armonk, New York, USA). The continuous variables were expressed as the mean and standard deviation. The *t*-test was utilized to compare between two groups when the two sets of data were in accordance with Gaussian distribution. Otherwise, the Wilcoxon test was performed for analysis. The categorical variables were presented as the absolute (no.) and relative (%) frequencies, and the chi-squared test was used to compare two groups. Significance level was set a = 0.05. Moreover, continuous and categorical variables were analyzed with ANOVA and chi-squared tests, respectively, to compare the three groups, whereas the significance level was adjusted as 0.0167 with Bonfferoni correction in chi-squared test for the comparison of three groups. The factors that possibly affected the intrapedicular accuracy and cranial FJV were also assessed with multivariate logistic regression analysis.

## Result

The mean age was 46.05 ± 12.73 years old, the gender ratio (F/M) was 27/47, and the average BMI was 22.88 ± 3.78 kg/m^2^. The baseline parameters showed no significant distances among the three groups in terms of the mean age, gender ratio, average BMI, and preoperative diagnosis (P > 0.05) (Table 1).

**Table 1:** Demographic data of study cohorts

Parameter	Overall	RA	FP	FH	P value
No. of patients	74	30	14	30	
Female, n (%)	27 (36.0)	10 (33.3)	8 (57.1)	8 (26.7)	0.138
Age (years)	46.05 ± 12.73	49.13 ± 10.19	41.64 ± 17.96	45.03 ± 11.80	0.129
BMI (kg/m^2^)	22.88 ± 3.78	22.99 ± 5.12	21.29 ± 2.21	23.50 ± 2.44	0.136
Preoperative diagnosis, n (%)					1.000
T11 fracture	5 (6.8)	2 (6.6)	1 (7.1)	2 (6.6)	
T12 fracture	20 (27.0)	8 (26.7)	4 (28.6)	8 (26.7)	
L1 fracture	34 (45.9)	14 (46.7)	6 (42.9)	14 (46.7)	
L2 fracture	15 (20.3)	6 (20.0)	3 (21.4)	6 (20.0)	
Level of screw instruments, n (%)					–
T10	14 (3.2)	2 (1.3)	2 (2.6)	10 (4.9)	
T11	58 (13.2)	14 (8.9)	10 (12.8)	34 (16.7)	
T12	113 (25.7)	44 (31.7)	22 (28.2)	47 (23.1)	
L1	120 (27.3)	54 (34.2)	20 (25.6)	46 (22.7)	
L2	88 (20.0)	36 (22.8)	12 (15.4)	40 (19.7)	
L3	36 (8.2)	8 (5.1)	8 (10.3)	20 (9.9)	
L4	10 (2.3)	0 (0)	4 (5.1)	6 (3.0)	

RA, robot-assisted; FP, fluoroscopy-guided percutaneous; FH, freehand; BMI, body mass index;

### Pedicle screw placement accuracy

The optimal intrapedicular accuracy of pedicle screw placement (Grade A) in the RA, FP, and FH groups was 94.3%, 78.2%, and 88.7%, respectively. This finding indicates no significant differences of RA over FH technique (P = 0.062) and FP technique (P = 0.025), but significantly higher accuracies of RA over FP (P < 0.001). In addition, the clinically acceptable accuracy of pedicle screw placement (Grade A + B) was 97.5%, 93.6%, and 97.0%, indicating no significant differences among the three techniques (P = 0.270, 1.000, 0.068) (Table 2).

**Table 2:** Pedicle screw placement accuracy among three insertion techniques

Screw position, n (%)	RA	FP	FH	P value^a^		
				RA vs FP	RA vs FH	FP vs FH
A	149 (94.3)	61 (78.2)	180 (88.7)	< 0.001	0.062	0.025
B	5 (3.2)	12 (15.4)	17 (8.4)	0.001	0.040	0.084
A + B	154 (97.5)	73 (93.6)	197 (97.0)	0.270	1.000	0.068
C	2 (1.3)	5 (6.4)	3 (1.5)	0.075	1.000	0.082
D	1 (0.6)	0 (0)	2 (1.0)	1.000	1.000	1.000
E	1 (0.6)	0 (0)	1 (0.5)	1.000	1.000	1.000
Total	158 (100)	82 (100)	203 (100)			

RA, robot-assisted; FP, fluoroscopy-guided percutaneous; FH, freehand

^a^The significance level was adjusted as 0.0167 with Bonfferoni correction

The adverse events among the three insertion techniques are displayed in Table 3. In RA, nine screws breached the cortexes of pedicle, with four screws and five screws penetrating medial and lateral cortexes, respectively. In FP, the most common deviation of screw in pedicle was medial in 10 pedicles (58.8%). Meanwhile, superior, inferior, and lateral deviations were observed in 0 (0), 2 (11.8%), and 5 (29.4%) pedicles, respectively. In FH, the most common deviation was also medial in 13 pedicles (56.5%). However, the remaining one, one, and eight screws caused superior, inferior, and lateral deviations, respectively. In addition, 0%, 7.1%, and 3.3% patients received intraoperative revision caused by screw malposition in the RA, FP, and FH groups, respectively, and no patient underwent postoperative revision caused by screw malposition or suffered from wound infections in the three groups.

**Table 3:** Adverse events among the three insertion techniques

Adverse events, n (%)	RA	FP	FH
Deviation of screw in pedicle			
Penetrations	9 (100)	17 (100)	23 (100)
Superior	0 (0)	0 (0)	1 (4.3)
Inferior	0 (0)	2 (11.8)	1 (4.3)
Medial	4 (44.4)	10 (58.8)	13 (56.5)
Lateral	5 (55.6)	5 (29.4)	8 (34.9)
Screw parts causing FJV			
Violations	22 (100)	24 (100)	46 (100)
Screw shaft	2 (9.1)	1 (4.2)	2 (4.3)
Screw head	20 (90.9)	23 (95.8)	43 (93.5)
Rod	0 (0)	0 (0)	1 (2.2)
Complications			
Patients	30 (100)	14 (100)	30 (100)
Intraoperative revision due to screw malposition	0 (0)	1 (7.1)	1 (3.3)
Postoperative revision due to screw malposition	0 (0)	0 (0)	0 (0)
Wound infections	0 (0)	0 (0)	0 (0)

### Proximal facet joint violation

The grades of proximal FJV are provided in Table 4. RA had a significantly greater proportion of intact facet joints than the FP (P = 0.002). However, FP and FH (P = 0.157), as well as RA and FH (P = 0.035) showed significantly similar outcomes with respect to the proximal FJV. Moreover, FP led to higher rates of severe FJV (grade 3) than RA (P = 0.004) and FH (P = 0.012), and RA was not superior to FH in severe FJV (P = 0.536). Furthermore, Table 3 shows that screw head resulted in most proximal FJVs in RA (90.9%), FP (95.8%), and FH (93.5%).

**Table 4:** Cranial facet joint violation among three insertion techniques

Cranial facet joint, n (%)	RA	FP	FH	P value^a^		
				RA vs FP	RA vs FH	FP vs FH
Grade 0	136 (86.1)	54 (69.2)	157 (77.3)	0.002	0.035	0.157
Grade 1	12 (7.6)	6 (7.7)	26 (12.8)	0.979	0.109	0.227
Grade 2	5 (3.2)	8 (10.3)	11 (5.4)	0.025	0.302	0.148
Grade 3	5 (3.2)	10 (12.8)	9 (4.4)	0.004	0.536	0.012
Total	158 (100)	82	203 (100)			

^a^The significance level was adjusted as 0.0167 with Bonfferoni correction

### Operation time and estimated blood loss

The comparison of time for operation and estimated blood loss in three techniques were evaluated as the average time per screw and average blood loss per screw because the number of inserted screws in different patients varied. We found that the RA (29.49 ± 5.82 min/screw), FP (27.60 ± 7.91 min/screw) and FH (28.86 ± 8.10 min/screw) techniques showed no significant differences in average time per screw (P > 0.05). Besides, FH (59.94 ± 26.49 ml/screw) resulted in significantly more blood loss per screw than RA (11.75 ± 2.20 ml/screw) and FP (11.61 ± 2.41 ml/screw), but no significant difference was observed in RA and FP techniques (P > 0.05).

### Factors associated with pedicle screw placement accuracy and cranial FJV

The logistic regression analyses showed that FP technique (OR 3.056, 95% Cl 1.129–8.269; P = 0.028) was independently associated with intrapedicular accuracy. Moreover, the age (OR 0.974, 95% Cl 0.955–0.994; P = 0.009), pedicle angle (OR 0.921, 95% Cl 0.859–0.988; P = 0.022), moderate facet joint osteoarthritis (OR 5.584, 95% Cl 2.100–14.850; P = 0.001), severe facet joint osteoarthritis (OR 11.956, 95% Cl 3.083–46.363; P < 0.001) were independently associated with cranial FJV (Table 5).

**Table 5:** Logistic regression analysis for screw misplacement

Variables	Intra-pedicular accuracy				Cranial facet joint violation			
	B	Odds ratio	95% Confidence intervals	P	B	Odds ratio	95% Confidence intervals	P
Age	− 0.012	0.988	(0.964, 1.012)	0.315	− 0.026	0.974	(0.955, 0.994)	0.009
Male gender	0.245	1.277	(0.617, 2.643)	0.510	–	–	–	–
BMI	− 0.055	0.946	(0.871, 1.027)	0.185	–	–	–	–
Pedicle screw placement techniques								
RA		1.000		0.079		–		
FP	1.117	3.056	(1.129, 8.269)	0.028	–	–	–	–
FH	0.738	2.091	(0.873, 5.008)	0.098	–	–	–	–
Pedicle angle	− 0.070	0.932	(0.856, 1.014)	0.103	− 0.082	0.921	(0.859, 0.988)	0.022
Instrumental levels								
0T10		1.000		0.378		–		
5T11	− 0.332	0.718	(0.146, 3.538)	0.684	–	–	–	–
6T12	− 0.820	0.440	(0.091, 2.129)	0.308	–	–	–	–
1L1	− 0.161	0.851	(0.178, 4.078)	0.840	–	–	–	–
2L2	− 0.341	0.711	(0.136, 3.714)	0.686	–	–	–	–
3L3	− 1.854	0.157	(0.013, 1.934)	0.148	–	–	–	–
4L4	0.609	1.839	(0.231, 14.653)	0.565	–	–	–	–
Facet joint osteoarthritis								
Normal		1.000		0.768		1.000		
Mild	− 0.435	0.647	(0.275, 1.523)	0.319	0.791	2.205	(0.898, 5.417)	0.085
Moderate	− 0.172	0.842	(0.302, 2.348)	0.743	1.720	5.584	(2.100, 14.850)	0.001
Severe	− 0.343	0.710	(0.143, 3.533)	0.675	2.481	11.956	(3.083, 46.363)	< 0.001
Distance from skin to insertion point	− 0.242	0.785	(0.477, 1.291)	0.340	0.113	1.119	(0.768, 1.631)	0.558

## Discussion

Pedicle screw placement has been widely used in spine surgery. It has gradually developed from free hand method in open surgery to percutaneous insertion in minimally invasive methods with different auxiliary equipment applied during the procedures of screw placement. However, in any case, the safety and accuracy of pedicle screw placement are essential. Thus, different instrumental techniques have been applied to increase intrapedicular accuracy and reduce cranial facet violation [6]. Recently, various robots including SpineAssist/Renaissance/Mazor X robots (Mazor Robotics Ltd., Caesarea, Israel), ROSA (Zimmer Biomet Robotics, Montpellier, France), and TiRobot system (TINAVI Medical Technologies Co. Ltd., Beijing, China) were designed to improve surgical accuracy and reduce radiation exposure [2, 12]. However, the outcomes concerning the superiority in intrapedicular accuracy and cranial facet joint protection of RA over FP and FH are conflicting [2, 6, 11–17]. The studies regarding TiRobot RA versus FP and FH are rare in previously published literature. Meanwhile, the thoracolumbar segments are the most common areas for spinal fractures and the main levels, where different pedicle screw placement techniques can be applied. Therefore, the study was designed to evaluate the rate and risk factors of the pedicle screw placement accuracy and cranial FJV between RA, FP, and FH techniques in thoracolumbar pedicle screw implantation.

For intrapedicular accuracy, Han et al. [12] and Feng et al. [9] conducted RCTs with TiRobot system. They concluded that the RA techniques were associated with significantly higher accuracy rates of 95.3% and 98.2% than the FH methods with the rates of 86.1% and 93.1%, respectively. Meanwhile, Yang et al. [11] revealed that Renaissance RA method showed remarkably increased inserting accuracy rate of 93.8% compared with the FP technique with the rate of 73.8%. However, some studies indicated that RA techniques performed no advantage over the conventional pedicle screw placement. Kim et al. [13] reported that no significant difference existed between Renaissance RA and FH groups regarding intrapedicular accuracy in an RCT, the outcome of which was similar to that in another prospective RCT conducted by Hyun et al. [14] Furthermore, Ringel et al. [15] found that the intrapedicular accuracy in FH was superior to that in RA technique. We have found in the present study that no significant differences existed between RA and FH techniques, and FP and FH techniques in terms of optimal accuracy, but a significantly lower rate of optimal accuracy of FP than RA was discernible. In addition, no remarkable differences were found among the three techniques in terms of the clinically acceptable accuracy. The results might be explained as follows. On the one hand, with the 3D images that provide more intuitive anatomical landmarks, the RA system can automatically formulate the optimal screw entry point and trajectory in accordance with the specific pedicle shape in different segments to reduce the manual errors and improve the insertion accuracy. Comparatively, although no auxiliary imaging was found in the FH method, the anatomical landmarks can be clearly revealed during the operation. Thus, the surgeons can identify the ideal entry point and insert screws through optimal trajectory in the surgical field of open version. Therefore, the RA and FH methods showed higher intrapedicular accuracy. However, when inserting the screws under fluoroscopy guidance in the FP method, the surgeon only relied on 2D images and limited tactile feedback to determine the entry point and trajectory. Meanwhile, the pedicle shadow was difficult to identify on the anteroposterior and lateral images, thereby easily causing the entry point of screws to the inside and reducing the abduction angle of the screw. As a result, the screws were more likely to penetrate the cortex of the pedicles in the FP technique compared with the RA and FH techniques.

Cranial FJV has been regarded as a crucial risk factor of ASD [3, 6, 8]. Moreover, the facet violation contributes to the relative displacement and angular deformity of the vertebrae, thereby resulting in the postoperative back pain and the instability of spine [4, 6]. Many studies have demonstrated that the FP technique showed higher rate of FJV than FH techniques [8, 18, 19, 28]. Babu et al. [19] compared the effects of FP versus FH on facet violation and found that 40.2% of the screws in FP group caused FJV. This finding was significantly higher than the 34.1% in FH group. Meanwhile, Teles et al. [8] performed a multivariate regression analysis showing that the risk of FJV caused by FP method was 3.31 times that of FH method. Moreover, the majority of literature reported that RA methods caused less cranial FJV than FP technique although both methods were percutaneous minimally invasive techniques [10, 13, 16, 18, 19]. Han et al. [12] performed an RCT with 1116 pedicle screws, revealing that none of the screws in the RA group violated the cranial facet joint. This finding was remarkably lower than that of 12 screws (2.1%) in the FH group. In addition, Yang et al. [11] and Archavlis et al. [7] found that RA showed evident advantages in facet joint protection with the rates of 94.9%–95.0% over FP with the rates of 78.0%–84.4%. Our comparative analysis revealed a significantly lower rate of cranial FJV in the RA group than in the FP group. However, no significant difference was found between the FP and FH methods as well as RA and FH methods.

The outcomes might be attributed to the following factors. The first factor is the guidance equipment, the selection of which can directly affect the clarity of the anatomical landmarks during the operation. This factor influences the rates of insertion accuracy and violation of facet joint. Different from the FP method mainly relying on the 2D images, the RA system can automatically identify the entry point and preplan insertion trajectory in accordance with the 3D images, which show anatomical structures clearly [21]. Thus, the screws easily pass through or rub the facet joints. The second factor is the resistance from soft tissues around the spine. RA and FP can protect the screws from strength of muscles with sleeves, and the pressure from soft tissue had a greater impact on FH, which affected the selection of entry point and direction of the trajectory. The entry point in the FH group tends to be inward, and the abduction angle is reduced. As a result, the FH technique performed no advantage over the RA technique; but it showed similar outcomes to the RA and FP technique. The third factor is the intrapedicular accuracy, where the RA methods performed well in the optimal implantation of pedicle screws according to the preplanned trajectory. As a result, the cranial FJV might be avoided as the preoperative planning. The fourth factor is the distance of pedicle screws from the facets. Kim et al. [13] reported that the mean distance of pedicle screws from facets in the FH group (2.7 ± 1.6 mm) was remarkably closer than in the RA group (5.2 ± 2.1 mm), indicating a lower possibility of the RA system in violating the cranial facet joints. The fifth factor is the learning curve effect. Kam et al. [29] found that RA pedicle screw placement had a very short (almost no) learning curve, showing that the RA technique was less demanding in surgical experience and skills of surgeon due to the automated and precise procedures. However, a higher level of skills is required for surgeons to increase the rate of intrapedicular accuracy and decrease the rate of FJV in the FH and FP methods.

The characteristics of the adverse events among the three techniques were reported. In the FH and FP groups, most deviations of screws were medial, thereby resulting in severe complications, such as spinal cord and nerve root injuries. However, in the RA group, most deviations were lateral, thereby reducing the possibility of severe consequences and revealing a safer choice for screw implantation. In addition, more than 90% cranial FJVs were caused by the screw head in three groups. This finding reminded the surgeons to insert screws in less depth on the premise to ensure spinal stability for facet joint protection. Moreover, for the complications, no patients underwent postoperative revision caused by screw malposition, and 3.3% and 7.1% of patients received intraoperative revision caused by screw malposition in the FH and FP groups. These results were higher than those of the RA group. Furthermore, no patients suffered from wound infections after surgery in three groups.

The factors that potentially affected the intrapedicular accuracy and cranial FJV need further investigation. Kim et al. [5] retrospectively evaluated 488 percutaneous pedicle screws in 110 consecutive patients and determined the obesity, measured by BMI, as the risk factor of screw malposition, because the obese or older patients would have hard muscles and higher resistance of soft tissues. Thus, the difficulty of selecting the best entry point and trajectory is increased [19, 30]. The statistical analysis also demonstrated that the FP pedicle screw placement technique was the risk factor for accurate insertion. The surgeons failed to accurately identify the landmarks with limited visual and tactile feedback, depending on the 2D images. Furthermore, we found that the facet osteoarthritis was the risk factor for cranial FJV due to the fact that the osteoarthritis of facet joints distorted the contour of the anatomical landmark in pedicle screw placement, thereby ultimately violating the cranial facet joint [31]. Even though the RA is safe and easy to handle, it still remains a procedure where breakdowns can occur. Meanwhile, the experience of the traditional techniques still remains very important. Furthermore the high costs of the RA still are a disadvantage which should be underlined.

The following limitations should be interpreted in this retrospective study. First, the clinical outcomes among the three techniques were not reported in the current study, and the relationship between the different grades of malposition and clinical outcomes need further investigation for the future work. Second, the clinical results were not discussed in this study, and the radiographic outcomes were obtained in short term after surgery without long-term follow-up. However, we assumed that the short-term results concerning the safety and accuracy of pedicle screw placement were crucial issues, thereby providing reference and reflection for surgeons. Third, the number of patients and screws included in the study is limited and the prospective trials with large sample size and high quality are needed in the future.

## Conclusion

RA technique showed a higher rate of intrapedicular accuracy and a lower rate of cranial FJV than FP technique, and similar outcomes to FH technique in terms of intrapedicular accuracy and cranial FJV. In addition, the FP technique was a risk factor for the intrapedicular accuracy. The RA technique might be a safe method for pedicle screw placement in spinal surgery.

## Data Availability

The datasets used and/or analyzed during the current study are available from the corresponding author on reasonable request.

## Abbreviations

FH: Freehand
FP: Fluoroscopy-guided percutaneous
RA: Robotic-assisted
FJV: Facet joint violation
RCTs: Randomized controlled trials
ASD: Adjacent segment degeneration
